# China's coordinated tripartite medical reform: strategic balancing of interests among pharmaceuticals, healthcare, and health insurance

**DOI:** 10.3389/fpubh.2025.1591358

**Published:** 2025-06-11

**Authors:** Hanxiang Gong, Wenbo Wu, Jifeng Li, Baoling Wu, Mengqi Gao, Yajun Yang

**Affiliations:** ^1^The Second Affiliated Hospital, Guangzhou Medical University, Guangzhou City, Guangdong, China; ^2^Faculty of Humanities and Social Sciences, Macau Polytechnic University, Macau, China

**Keywords:** healthcare reform, evolutionary game theory, pharmaceutical policy, medical insurance regulation, stakeholder coordination

## Abstract

China has implemented the “Triple-Medical” reform, aiming to enhance the overall efficacy of the public healthcare system through deep integration and coordination among healthcare, pharmaceuticals, and health insurance regulator. This study utilizes game theory to analyze the strategic interplay and conflicts of interest among these three sectors within China's reform context, exploring the strategic choices and interactions that occur during the reform process. This study utilizes game theory to analyze the strategic interplay among pharmaceutical companies, healthcare institutions, and the health insurance regulator in China's healthcare reform. The model examines key variables and behaviors of each stakeholder, with MATLAB simulations analyzing evolutionarily stable strategies and parameter sensitivity. The findings reveal complex dynamic interactions among the strategies adopted by the various stakeholders within the healthcare reform, with the optimal strategies converging at the equilibrium point. Specifically, pharmaceutical companies seek maximum economic gains through drug pricing and quality control; healthcare institutions strive to enhance service efficiency and quality to meet patient needs; and medical insurance regulatory bodies play a crucial role in ensuring the efficiency and fairness of fund utilization. Such strategic alignments contribute to the stable operation of the healthcare system and maximize the interests of all parties involved. The study concludes that coordinated strategies among pharmaceutical companies, healthcare institutions, and health insurance regulator can achieve equilibrium and enhance the efficiency and equity of China's healthcare system. Changes in penalties for pharmaceutical companies, costs of medical service quality, and medical insurance regulatory costs critically influence healthcare reform, providing empirical support and a theoretical basis for effective policy-making. Refining policy adjustments and strategic optimizations can effectively coordinate the interests of all parties, propelling China's healthcare system toward greater efficiency and fairness.

## 1 Introduction

Globally, healthcare reform remains a central issue for policymakers and the public alike. With technological advancements and the progression of population aging, the healthcare sector faces challenges such as improving the quality of medical services, ensuring equitable distribution of healthcare resources, and addressing rising healthcare costs (1, 2). Healthcare reform is a common dilemma worldwide, with its complexity stemming from the need to balance enhancing service quality, ensuring accessibility, and reducing costs. Due to cultural, political, and economic differences, countries adopt varied strategies and measures in their healthcare reforms. Both developing and developed nations strive to use various means and policies to optimize the quality, accessibility, and efficiency of medical services. The challenge of healthcare reform lies in finding a precise balance between maintaining service quality, curbing the growth of medical expenditures, and fairly distributing healthcare resources. Although specific socio-economic conditions and reform challenges differ from country to country, healthcare reform undoubtedly represents a significant issue involving public health policy, social equity, and economic efficiency.

In recent years, China has actively implemented the “Triple-Medical Linkage” reform strategy, aimed at comprehensively advancing the reform of the healthcare system through deep integration and coordination among the healthcare, pharmaceutical, and health insurance regulator (3–7). This strategy focuses on optimizing policies and mechanisms to enhance the efficiency of medical services, reduce healthcare costs, and strengthen the overall efficacy of the public healthcare system (8–11). This reform is guided by several key policy documents, including *the National Pilot Plan for Drug Centralized Procurement and Use (State Council General Office, Document No. [2019] 2)*, which launched volume-based procurement as a tool to regulate drug pricing, and the *Guiding Opinions on Further Deepening the Reform of Basic Medical Insurance Payment Methods (National Healthcare Security Administration, Document No. [2021] 37)*, which promotes payment methods such as DRGs and per capita payment. Measures within the “Triple-Medical Linkage” include optimizing the distribution of medical resources, enhancing the capabilities of primary healthcare institutions, promoting contracted family doctor services, and achieving continuous and coordinated medical services through informatization. The reform also addresses the drug supply chain and pricing mechanisms by implementing centralized drug procurement, enforcing zero-markup policies for hospital drug sales, and strengthening the regulation of the pharmaceutical market to ensure drug quality and supply safety. Additionally, reforms in the medical insurance system aim to refine payment methods, promote disease-based and per capita payment models, and strengthen the supervision of medical insurance funds to expand coverage and increase reimbursement rates. Through these comprehensive reform measures, the “Triple-Medical Linkage” strives to build an efficient and equitable healthcare service system.

However, the implementation of the “Triple-Medical Linkage” faces numerous challenges: Primary healthcare institutions are hampered by uneven resource distribution and shortages of equipment and personnel, which limit the enhancement of service capabilities. The pharmaceutical industry resists drug price controls and supply chain reforms, impacting the implementation of policies. In medical insurance reform, the introduction of new payment methods, such as disease-based payments, is difficult to apply universally, potentially leading to mismanagement and wastage of funds. An incomplete regulatory mechanism increases the risks of resource waste and fluctuations in medical quality. Therefore, to ensure the successful implementation of the “Triple-Medical Linkage” policy, it is imperative to address the uneven distribution of resources, resistance from the pharmaceutical industry, challenges in implementing new medical insurance payment methods, and the strengthening of policy regulatory mechanisms, thereby safeguarding the quality and efficiency of medical services and achieving long-term sustainable healthcare system reform.

This study establishes a game theory model to simulate the strategic interactions among pharmaceutical companies, healthcare institutions, and medical insurance regulatory bodies in China's healthcare reform. Through parameter analysis and sensitivity testing, it reveals how different strategic choices can achieve equilibrium in strategic adjustments and synergistic effects, and how these choices impact the effectiveness of healthcare reform. The significance of this research lies in dissecting the interactions and influences among the three parties, which helps identify key factors in the reform and provides viable recommendations for future policy-making. The novelty of this paper is in utilizing evolutionary game theory to comprehensively analyze the mutual impacts among reform stakeholders, offering a new method of understanding the complexity and dynamics of China's healthcare reform.

Specifically, the core objectives of this study are to: (1) develop a comprehensive game-theoretical model that captures the strategic interactions among pharmaceutical companies, healthcare institutions, and health insurance regulatory agencies in the context of China's healthcare reform; (2) identify and analyze the key variables and critical factors that drive these interactions; (3) determine the conditions under which equilibrium strategies can be achieved to maximize the interests of all stakeholders; and (4) conduct extensive sensitivity analysis using MATLAB simulations to evaluate the impact of parameter variations on system stability, thereby providing empirical support and theoretical guidance for effective policy-making in healthcare reform. This study advances the existing literature in three key ways. First, unlike prior studies that typically examine dyadic interactions (e.g., insurer–provider or hospital–pharma), this paper introduces a tripartite evolutionary game model that reflects the real-world complexity of China's reform landscape. Second, the integration of replicator dynamics and parameter sensitivity analysis enables a dynamic, non-linear exploration of strategic adaptation, going beyond static or descriptive models. Third, the model incorporates specific institutional features of China's “Triple-Medical Linkage” reform, making it one of the few studies to provide both theoretical rigor and strong policy relevance tailored to the Chinese context.

Compared with classical game theory, evolutionary game theory offers a more realistic and flexible framework for analyzing stakeholder behavior in healthcare reform. Traditional game theory typically assumes complete rationality and static or repeated interactions under full information, which limits its explanatory power in complex, regulated environments such as healthcare systems. In contrast, evolutionary game theory is based on the assumption of bounded rationality, reflecting how healthcare providers, pharmaceutical firms, and insurance regulators adapt strategies gradually over time under incomplete information and policy constraints. This aligns well with the institutional realities in China, where decisions are often shaped by regulatory cycles, performance indicators, and asymmetric access to data. Recent studies have further validated the applicability of evolutionary game models in healthcare policy design. For instance, Wang et al. (12) applied evolutionary dynamics to explore strategy diffusion in face-swiping medical services under rationality limits of providers and users. Yue et al. (13) developed a public-private partnership model for elderly healthcare using evolutionary game theory, demonstrating its robustness under information asymmetry. These findings support the theoretical and practical relevance of our chosen framework in capturing dynamic strategic adjustments under policy-driven constraints.

## 2 Literature review

In response to the growing demand for healthcare services, governments worldwide have conducted extensive and deep reforms of their healthcare systems (14–18). Globally, healthcare reforms largely aim to control healthcare costs to maintain their sustainability within public budgets (19, 20). Simultaneously, enhancing the quality of medical services, reducing medical errors, and increasing patient satisfaction are also primary objectives of healthcare reforms in various countries (21–23). Some developed countries, such as the United States, have attempted to reform healthcare through measures such as increasing insurance coverage, modifying payment systems, and advancing medical technology innovations (24–26). In developing countries, due to limited resources, reforms often focus on optimizing resource allocation, strengthening public health services, and improving basic medical facilities (27–32). Although each country's healthcare system and path of reform differ, they all share a common goal: to find an ideal balance between healthcare quality, accessibility, and affordability.

China's healthcare reform dates back to the 1980s. Initially, the government promoted market-oriented reforms, encouraging healthcare institutions to be financially self-sufficient, which led to a sharp increase in medical service costs (33–35). In recent years, healthcare reform has unfolded among pharmaceutical companies, healthcare institutions, and medical insurance regulatory bodies. The government is committed to addressing medical issues arising from supply-demand imbalances and regional and economic disparities, such as attempting to lower the cost of healthcare services, improve medical insurance payment methods, and reform healthcare service provision (36–38). However, the process of healthcare reform has not been smooth. Due to the problem of interest distribution, the interactions among pharmaceutical companies, healthcare institutions, and medical insurance regulatory bodies are highly complex. Reformers face significant challenges in balancing cost reduction and quality enhancement, and in reforming the allocation of medical resources and the medical insurance system to make healthcare services more equitable. Key healthcare reform policies in China are shown in Table 1.

**Table 1:** Key healthcare reform policies in China.

**Reform phase**	**Timeframe**	**Key policy and relevant document**	**Policy significance**
Phase 1—Introduction of “Pharmaceutical-Led Healthcare” Model	1980–1989	Introduction of the “PharmaceuticalLed Healthcare” Model.	Introduction of the pharmaceutical sales as the primary source of revenue for healthcare institutions, encouraging pharmaceutical companies to produce and sell drugs.
		Establishment of rural cooperative medical care.	Provision of basic medical insurance for rural residents, promoting the development of rural healthcare.
Phase 2—Advancement of Urban and Rural Healthcare Insurance Systems	1990–1999	Pharmaceutical price reforms and the “Southern Medicine Price Storm”.	Controlling drug price increases and enhancing drug price transparency through pharmaceutical price reforms.
		Launch of the Urban Residents Basic Medical Insurance (URBMI) pilot program.	Expansion of healthcare coverage and improvement in the healthcare security for urban residents.
		Establishment of the New Rural Cooperative Medical Care System (NRCMCS).	Strengthening the NRCMCS to improve healthcare coverage for rural residents.
Phase 3—Strengthening Public Health and Basic Medicines	2000–2009	Outbreak of SARS epidemic raises public health concerns.	Enhancement of the public health system and improvement of the capacity to respond to public health emergencies.
		Introduction of the Basic Medicine System.	Ensuring the supply and reasonable pricing of essential medicines to meet public demand.
Phase 4—Comprehensive Reform of the Medical and Healthcare System	2010–2018	Comprehensive reform of the medical and healthcare system.	Promotion of comprehensive reform of the medical and healthcare system to improve healthcare service quality and efficiency.
		Integration of urban and rural resident health insurance schemes and expansion of insurance coverage.	Integration of urban and rural health insurance systems, expanding coverage, and reducing the financial burden of medical expenses.
		Release of the “Healthy China 2030” plan.	Establishment of the Healthy China 2030 goals, aiming for the wellbeing and comprehensive development of the population.
Phase 5—New Healthcare Reform and Adjustment of Healthcare Insurance Policies	2019	Focus on addressing unequal access to healthcare services and high drug prices in the new healthcare reform.	Optimization of healthcare resource allocation, enhancing equity and accessibility of healthcare services.
		Adjustment of urban and rural resident healthcare insurance policies.	Policy adjustments to reduce the financial burden of healthcare expenses for residents.
		Expansion of the National Essential Medicines List.	Expansion of the range of essential medicines, ensuring their supply.
Phase 6—Coping with the COVID-19 Pandemic and Strengthening the Healthcare System	2020–2022	Impact of the COVID-19 pandemic on the healthcare system.	Enhancement of the capacity to respond to public health emergencies, ensuring public health safety.
		Strengthening the construction of the public health system.	Improvement of basic public health services, strengthening epidemic monitoring, and prevention.
		Expansion of healthcare insurance coverage.	Expansion of healthcare expense reimbursement coverage, reducing the economic burden on patients.
		Promotion of the Global Health 2030 Initiative.	Participation in global health initiatives, promoting sustainable global health development.
Phase 7—Post-Pandemic Era	2023 to Present	Strengthening disaster response capabilities of the healthcare system.	Enhancement of the healthcare system's disaster response capabilities to address future public health emergencies.
		Promotion of digital healthcare services development.	Advancement of digital healthcare services to improve healthcare efficiency and convenience.
		Reinforcement of healthcare insurance sustainability.	Ensuring the sustainability of the healthcare insurance system, maintaining healthcare security stability.

In the process of healthcare reform in China, the strategic interactions among pharmaceutical companies, healthcare institutions, and medical insurance regulatory bodies play a critical role. The dynamics of these interactions often determine the effectiveness of healthcare policy implementation and influence the progress and direction of healthcare reforms. For instance, pharmaceutical companies negotiate with government insurance departments over drug pricing and supply, while healthcare institutions engage in negotiations regarding the provision and quality of healthcare services. However, healthcare reform is not merely a game among these three parties; it also requires their cooperation. During the formulation and implementation of healthcare policies, pharmaceutical companies, healthcare institutions, and medical insurance regulatory bodies need to collaborate and coordinate to develop joint strategies. They must find common ground while engaging in strategic negotiations to ensure the smooth progress of healthcare reform.

Although global research has provided profound insights into healthcare reform strategies, including cost control, quality improvement, and the expansion of medical coverage, current literature still lacks a systematic analysis of the dynamics of interactions among medical, pharmaceutical, and insurance sectors and their impact on policy-making. Particularly, although the Chinese government has implemented a series of reforms to optimize interactions among these three sectors, there is a paucity of research on finding sustainable and effective mechanisms for collaboration among different stakeholders. Therefore, this study constructs a game theory model to explore the strategic choices and interactive effects among the participants in the tripartite healthcare reform process (39–41), thereby proposing more specific and operational policy recommendations to advance healthcare reforms toward a deeper and more detailed direction.

Compared with global reforms, recent China-specific studies—such as those on volume-based procurement and DRG payment pilots—highlight the systemic impact of integrated policies. These reforms have reduced drug prices, improved cost-efficiency, and strengthened cross-sector collaboration. This study builds on such localized frameworks by embedding these dynamics into an evolutionary game model, offering a unified perspective on tripartite strategic coordination.

## 3 Model description and basic assumptions

### 3.1 Model description

This paper delves into the healthcare reform in China from the perspective of the evolutionary game among pharmaceutical companies, healthcare institutions, and medical insurance regulatory bodies. In this study, these three stakeholders are considered key players in the game, and a series of variables are designed to characterize their behavior and decision-making. Below are all the variables used in the model and their meanings

Firstly, from the perspective of pharmaceutical companies, the following key variables are considered. *M*_1_ represents the reasonable sales revenue of drugs, which reveals the economic returns a company can obtain from regulated and legal drug sales. *M*_2_ denotes the unreasonable sales revenue of drugs, reflecting the higher profits that might be achieved through non-standard practices. *C*_*d*_ and *C*_*s*_ represent the costs of research and development and drug quality and safety, respectively, illustrating the company's investment in developing new drugs and ensuring drug safety. Finally, *F*_*m*_ refers to the fines imposed by the government on pharmaceutical companies, reflecting the punitive measures that might be taken when companies violate regulations.

Secondly, from the perspective of healthcare institutions, the following key variables are considered. *W*_1_ represents the revenue obtained from providing high-quality medical services, while *W*_2_ represents the revenue from low-quality services. These variables reflect the disparity in earnings that healthcare institutions experience under different levels of service quality. *C*_*q*_ denotes the cost of service quality, indicating the resources that healthcare institutions need to invest to provide high-quality services. *S* points to additional government subsidies, revealing how the government motivates healthcare institutions to improve their services through financial support. *F*_*h*_ is the fine imposed by the health insurance regulator on healthcare institutions, reflecting the constraints and control exerted by the health insurance sector.

Lastly, from the perspective of the health insurance regulatory department, the following key variables are set. *P* represents the prepayment from the health insurance fund, reflecting the funds advanced by the health insurance department to ensure the provision of medical services. *I* denotes the income of the health insurance fund, expressing the total funds acquired by the health insurance department through various means. *Y*_*b*_ and *F*_*b*_, respectively, represent the reward and penalty mechanisms, showcasing how the health insurance department influences the behavior of healthcare institutions and pharmaceutical companies through incentives and penalties. Lastly, *C*_*r*_ and *C*_*g*_ are the costs of rectification and regulation, respectively; these variables reveal the resources required by the health insurance department in the execution of its duties.

Amid the advancement of healthcare reform in China, a complex interplay unfolds among pharmaceutical companies, healthcare institutions, and health insurance regulator. Throughout this interaction, each party pursues its interests and objectives, yet is influenced by the actions of the others, necessitating a balancing of various decisions. Firstly, pharmaceutical companies, as producers and sellers of medications, must consider health insurance policies and the demands of healthcare institutions when setting drug pricing strategies. Healthcare institutions face the decision to become designated hospitals under health insurance schemes. Being designated provides additional financial support from the health insurance sector but also requires adherence to specific regulations and policies. Finally, the health insurance regulator act as regulators, responsible for formulating health insurance policies and overseeing their implementation. They can choose to provide financial subsidies to encourage healthcare institutions to offer high-quality services or to impose penalties for non-compliance to maintain the stability of the health insurance system. In China's institutional context, the behavior of both healthcare institutions and pharmaceutical companies is shaped not only by market incentives but also by policy instruments, such as fiscal subsidies for public hospitals and volume-based procurement for pharmaceuticals.

For simulation purposes, the quantitative settings of these variables were informed by relevant policy documents, empirical references, and parameterized assumptions that reflect the reform context in China. A summary of their structural roles and sources has been considered to ensure internal consistency and conceptual transparency.

### 3.2 Model assumptions

To construct the game-theoretical model and analyze the stability of equilibrium points and the interrelationships among variables, the following assumptions are made:

Assumption 1: Decision-making behaviors of pharmaceutical companies, healthcare institutions, and health insurance regulator are all based on rational choices, aimed at optimizing their own interests.
Assumption 2: The decisions of pharmaceutical companies, healthcare institutions, and health insurance regulator influence each other. For instance, the revenue decisions from pharmaceutical sales will affect the quality of services offered by healthcare institutions, which in turn influences the reward and penalty decisions of the health insurance department.
Assumption 3: Pharmaceutical companies aim to maximize their revenue, which includes profits from drug sales (both legitimate and illegitimate) while striving to minimize research and development costs and costs related to drug quality and safety. Penalties imposed by the government are costs that these companies seek to avoid. However, under policies such as Volume-based Procurement, the room for excessive pricing and abnormal profit margins (*M*_2_) is strictly constrained, thus reshaping their strategic space.
Assumption 4: The primary goal of healthcare institutions is to optimize the ratio of their revenues to costs, including maximizing revenues from high-quality medical services and subsidies from the government, while minimizing the costs associated with service quality. Penalties imposed by the health insurance department are costs that these institutions seek to avoid. Considering the public welfare nature of public hospitals in China, the strategic choices of healthcare institutions are not solely driven by profit maximization but must also balance the synergistic relationship between high-quality service revenue (*W*_1_) and government fiscal subsidies (*S*), which jointly affect their incentive structures.
Assumption 5: The main task of the health insurance department is to maintain the balance of the health insurance fund by effectively managing prepayments and income, implementing effective reward and penalty mechanisms, and minimizing rectification and regulatory costs.
Assumption 6: The reward and penalty mechanisms imposed by the government on pharmaceutical companies and healthcare institutions effectively regulate their behavior. Fines effectively prevent unreasonable actions, while subsidies and rewards incentivize compliant behavior. Pharmaceutical companies, healthcare institutions, and health insurance regulator will gradually adjust their strategies based on the actions of others and their own experiences, thus evolving the dynamics of the game.

### 3.3 Model construction

In the game-theoretic model, let *x* represent the proportion of pharmaceutical companies producing and selling drugs at reasonable prices, while 1−*x* represents the proportion of those selling at unreasonable prices. Let *y* denote the proportion of patients accepting medical treatment, and 1−*y* he proportion of patients refusing treatment. Let *z* denote the willingness of health insurance regulator to enforce strict regulations, while 1−*z* represents their inclination toward lenient regulation. It is assumed that *x, y, z*∈(0, 1).Based on these assumptions and variable definitions, a mixed-strategy game payoff matrix involving healthcare institutions, patients, and health insurance regulator is constructed as shown in Table 2.

**Table 2:** Mixed-strategy game matrix of pharmaceutical companies, healthcare institutions and health insurance regulator.

**Stakeholders/Actions**	**Healthcare institutions**		**Health insurance regulator**	
			**Strict regulation (** ***z*** **)**	**Lax regulation (1 –** ***z*****)**
Pharmaceutical manufacturing and distribution companies	Production and distribution of reasonably priced medicines (*x*)	High-quality services (*y*)	*M*_1_−*C*_*d*_−*C*_*s*_ *W*_1_−*C*_*q*_+*S*+*P* I – *P* + *Y*_*b*_−*C*_*r*_−*C*_*g*_−*S*	*M*_1_−*C*_*d*_−*C*_*s*_ *W*_1_−*C*_*q*_+*S*+*P* *I*−*P*−*C*_*r*_−*C*_*g*_−*S*
		Low-quality services (1 – *y*)	*M*_1_−*C*_*d*_−*C*_*s*_ *M*_2_+*P*−*C*_*q*_−*F*_*h*_ *I*−*P*+*Y*_*b*_−*C*_*r*_−*C*_*g*_+*F*_*h*_	*M*_1_−*C*_*d*_−*C*_*s*_ *M*_2_+*P*−*C*_*q*_ *I*−*P*
	Production and distribution of unreasonably priced medicines (1 – *x*)	High-quality services (*y*)	*M*_2_−*C*_*d*_−*C*_*s*_−*F*_*m*_ *W*_1_−*C*_*q*_+*S*+*P* *I*−*P*+*Y*_*b*_−*C*_*r*_−*C*_*g*_+*F*_*m*_	*M*_2_−*C*_*d*_−*C*_*s*_ *W*_1_−*C*_*q*_+*S*+*P* *I*−*P*
		Low-quality services (1 – *y*)	*M*_2_−*C*_*d*_−*C*_*s*_−*F*_*m*_ *M*_2_+*P*−*C*_*q*_−*F*_*h*_ *I*−*P*+*Y*_*b*_−*C*_*r*_−*C*_*g*_+*F*_*m*_+*F*_*h*_	*M*_2_−*C*_*d*_−*C*_*s*_ *M*_2_+*P*−*C*_*q*_ *I*−*P*

### 3.4 Replicator dynamics equations

#### 3.4.1 Pharmaceutical companies

Profit from producing and selling drugs at reasonable prices:


$$\begin{array}{l} \;E_{x}=(M_{1}-C_{d}-C_{s})*y*z+(M_{1}-C_{d}-C_{s})*y*(1-z)\\ +(M_{1}-C_{d}-C_{s})*(1-y)*z+(M_{1}-C_{d}-C_{s})*(1-y)*(1-z) \end{array}$$

Profit from producing and selling drugs at unreasonable prices:


$$\begin{array}{l} E_{1-x}=\left(M_{2}-C_{d}-C_{s}-F_{m}\right)*y*z+\left(M_{2}-C_{d}-C_{s}\right)*\\ y*\left(1-z\right)+\left(M_{2}-C_{d}-C_{s}-F_{m}\right)*\left(1-y\right)*z\\ +\left(M_{2}-C_{d}-C_{s}\right)*\left(1-y\right)*\left(1-z\right) \end{array}$$

Average profit for pharmaceutical companies:


$$\begin{array}{l} \bar{E}=xE_{x}+\left(1-x\right)E_{1-x} \end{array}$$

Replicator dynamics equation for pharmaceutical companies:


$$\begin{array}{l} F\left(x\right)=-x*\left(x\;-\;1\right)*\left(M_{1}\;-\;M_{2}\;+\;F_{m}*z\right) \end{array}$$

#### 3.4.2 Healthcare institutions

Profit from providing high-quality services:


$$\begin{array}{l} E_{y}=\left(W_{1}-C_{q}+S+P\right)*x*z+\left(W_{1}-C_{q}+S+P\right)*\\ x*\left(1-z\right)+\left(1-x\right)*z*\left(W_{1}-C_{q}+S+P\right)\\ +\left(W_{1}-C_{q}+S+P\right)*\left(1-x\right)*\left(1-z\right) \end{array}$$

Profit from providing low-quality services:


$$\begin{array}{l} E_{1-y}=\left(M_{2}+P-C_{q}-F_{h}\right)*x*z+\left(M_{2}+P-C_{q}\right)*\\ x*\left(1-z\right)+\left(1-x\right)*z*\left(M_{2}+P-C_{q}-F_{h}\right)\\ +\left(M_{2}+P-C_{q}\right)*\left(1-x\right)*\left(1-z\right) \end{array}$$

Average profit for healthcare institutions:


$$\begin{array}{l} \bar{E}=yE_{y}+\left(1-y\right)E_{1-y} \end{array}$$

Replicator dynamics equation for healthcare institutions:


$$\begin{array}{l} F\left(y\right)=-y*\left(y\;-\;1\right)*\left(S\;-\;M_{2}\;+W_{1}\;+\;F_{h}*z\right) \end{array}$$

#### 3.4.3 Health insurance regulator

Profit from strict regulation:


$$\begin{array}{l} E_{z}=\left(I-P+Y_{b}-C_{r}-C_{g}-S\right)*x*y\\ +\left(I-P+Y_{b}-C_{r}-C_{g}+F_{h}\right)*x*\left(1-y\right)\\ +\left(I-P+Y_{b}-C_{r}-C_{g}+F_{m}\right)*\left(1-x\right)*y\\ +\left(I-P+Y_{b}-C_{r}-C_{g}+F_{m}+F_{h}\right)*\left(1-x\right)*\left(1-y\right) \end{array}$$

Profit from lenient regulation:


$$\begin{array}{l} E_{1-z}=\left(I-P-C_{r}-C_{g}-S\right)*x*y+\left(I-P\right)*x*\left(1-y\right)\\ +\left(I-P\right)*\left(1-x\right)*y+\left(I-P\right)*\left(1-x\right)*\left(1-y\right) \end{array}$$

Average profit for health insurance regulator:


$$\begin{array}{l} \bar{E}=zE_{z}+\left(1-z\right)E_{1-z} \end{array}$$

Replicator dynamics equation for health insurance regulator:


$$\begin{array}{l} F(z)=-z*(z\;-\;1)*(F_{h}\;-\;C_{r}\;-\;C_{g}\;+\;F_{m}\;+Y_{b}\\ \;-\;F_{m}*x\;-\;F_{h}\;*y\;+\;C_{g}*x*y\;+\;C_{r}*x*y) \end{array}$$

### 3.5 Stability analysis and equilibrium points

The interactions among pharmaceutical companies, healthcare institutions, and health insurance regulator are constantly evolving, meaning that the probability of any strategy selected by the parties is time-dependent. According to the principles of differential equation stability, when all the dynamic equations equate to zero, it indicates that the entire dynamic system will tend toward stability. The equilibrium points of the tripartite evolutionary game can be determined by setting *F*(*x*) = 0, *F*(*y*) = 0, *F*(*z*) = 0. It follows that:


$$\begin{array}{l} \;F\left(x\right)=-x*(x\;-\;1)*(M_{1}\;-\;M_{2}\;+\;F_{m}*z)=0\\ F\left(y\right)=-y*(y\;-\;1)*(S\;-\;M_{2}\;+W_{1}\;+\;F_{h}*z)=0\\ \;F\left(z\right)=-z*(z\;-\;1)*(F_{h}\;-\;C_{r}\;-\;C_{g}\;+\;F_{m}\;+Y_{b}\\ \;-\;F_{m}*x\;-\;F_{h}\;*y\;+\;C_{g}*x*y\;+\;C_{r}*x*y)=0 \end{array}$$

There are evidently eight distinct equilibrium points *E*_1_(0, 0, 0), *E*_2_(1, 0, 0), *E*_3_(0, 1, 0), *E*_4_(0, 0, 1), *E*_5_(1, 1, 0), *E*_6_(1, 0, 1), *E*_7_(0, 1, 1), *E*_8_(1, 1, 1), where all stakeholders adopt pure strategies at each equilibrium point.

Based on the replicator dynamics equations of the three parties, the Jacobian matrix of the evolutionary game system can be derived as follows:


$$\begin{array}{l} J=\left[\begin{array}{ccc} \frac{\partial F(x)}{\partial x} & \frac{\partial F(x)}{\partial y} & \frac{\partial F(x)}{\partial z}\\ \frac{\partial F(y)}{\partial x} & \frac{\partial F(y)}{\partial y} & \frac{\partial F(y)}{\partial z}\\ \frac{\partial F(z)}{\partial x} & \frac{\partial F(z)}{\partial y} & \frac{\partial F(z)}{\partial z} \end{array}\right]=\left[\begin{array}{ccc} J_{11} & J_{12} & J_{13}\\ J_{21} & J_{22} & J_{23}\\ J_{31} & J_{32} & J_{33} \end{array}\right]\\ J_{11}=\;-\;x*(M_{1}\;-\;M_{2}\;+\;F_{m}*z)\;-\;(x\;-\;1)*\\ \left(M_{1}\;-\;M_{2}\;+\;F_{m}*z\right)\\ J_{12}=0\\ J_{13}=\;-F_{m}*x*(x\;-\;1)\\ J_{21}=0\\ J_{22}=-\;(y\;-\;1)*(S\;-\;M_{2}\;+W_{1}\;+\;F_{h}*z)\\ -\;y*(S\;-\;M_{2}\;+W_{1}\;+\;F_{h}*z)\\ J_{23}=\;-Fh*y*(y\;-\;1)\\ J_{31}=-z*(z\;-\;1)*(C_{g}*y\;-\;F_{m}\;+\;C_{r}*y)\\ J_{32}=-z*(z-1)*(C_{g}*x\;-\;F_{h}\;+\;C_{r}*x)\\ J_{33}=-z*(F_{h}\;-\;C_{r}\;-\;C_{g}\;+\;F_{m}\;+Y_{b}\;-\;F_{m}*x\;-\;F_{h}\;*y\;+\\ C_{g}*x*y\;+\;C_{r}*x*y)-(z-1)*(F_{h}\;-\;C_{r}\\ -\;C_{g}\;+\;F_{m}\;+Y_{b}\;-\;F_{m}*x\;-\;F_{h}\;*y\;+\;C_{g}*x*y\;+\;C_{r}*x*y) \end{array}$$

While this study primarily analyzes pure strategy equilibrium points for simplicity and interpretability, it is worth noting that in real-world healthcare systems, stakeholders often adopt mixed strategies—especially under uncertain regulatory environments or fluctuating market conditions. For instance, pharmaceutical companies may probabilistically adjust their pricing schemes depending on regulatory signals, and healthcare institutions may vary service quality depending on patient demographics or resource constraints. Therefore, the model could be further extended in future studies to examine the existence and stability of mixed-strategy equilibria, offering a more nuanced understanding of strategic behaviors in healthcare reform.

### 3.6 Equilibrium solutions and evolutionarily stable strategies

The interactions among pharmaceutical companies, healthcare institutions, and health insurance regulator are constantly evolving, meaning that the probability of any strategy selected by the parties is time-dependent. According to the principles of differential equation stability, when all the dynamic equations equate to zero, it indicates that the entire dynamic system will tend toward stability. The equilibrium points of the tripartite evolutionary game can be determined by setting *F*(*x*) = 0, *F*(*y*) = 0, *F*(*z*) = 0.

There are evidently eight distinct equilibrium points *E*_1_(0, 0, 0), *E*_2_(1, 0, 0), *E*_3_(0, 1, 0), *E*_4_(0, 0, 1), *E*_5_(1, 1, 0), *E*_6_(1, 0, 1), *E*_7_(0, 1, 1), *E*_8_(1, 1, 1), where all stakeholders adopt pure strategies at each equilibrium point.

Based on the replicator dynamics equations of the three parties, the Jacobian matrix of the evolutionary game system can be derived to assess the stability of these equilibrium points. There may exist two evolutionarily stable equilibrium points in the evolutionary game system: For *E*_8_(1, 1, 1) to be an equilibrium point, the stability conditions *M*_2_ − *M*_1_ − *F*_*m*_ < 0 and *M*_2_− *F*_*h*_ − *S* − *W*_1_ < 0 must be satisfied.

Using the Lyapunov method, it is known that in the stability analysis of differential systems, stability can be assessed based on the sign of the characteristic roots at equilibrium points. If all characteristic values (roots) at an equilibrium point are negative, the point is considered an evolutionarily stable strategy (asymptotically stable point). The characteristic values for each of the eight pure strategy points are obtained by substituting these points sequentially into the Jacobian matrix.

As shown in Table A, there may exist two evolutionarily stable equilibrium points in the evolutionary game system: For *E*_8_(1, 1, 1) to be an equilibrium point, the stability conditions *M*_2_-*M*_1_-*F*_*m*_ < 0 and *M*_2_-*F*_*h*_-S-*W*_1_ < 0 must be satisfied.

## 4 Numerical simulation analysis

Using the MATLAB software, this paper conducts simulation analyses of evolutionarily stable strategies and their sensitivity to parameters, based on the results from the game-theoretical model described in Section 4.

### 4.1 Evolutionarily stable strategies

To make the simulation process more understandable for readers who may not have a technical background in game theory, we briefly outline the simulation logic used in MATLAB. First, initial strategy proportions for the three stakeholder groups—pharmaceutical companies, healthcare institutions, and health insurance regulators—are defined based on realistic assumptions. Then, we apply replicator dynamics equations to simulate how these strategies evolve over time through iterative updates. The simulation tracks the probabilities of each group adopting specific strategies (e.g., reasonable pricing, high-quality services, strict regulation) across 50 iterations. The goal is to observe whether the system converges to a stable equilibrium, and under what parameter conditions this convergence occurs.

**Figure 1:**
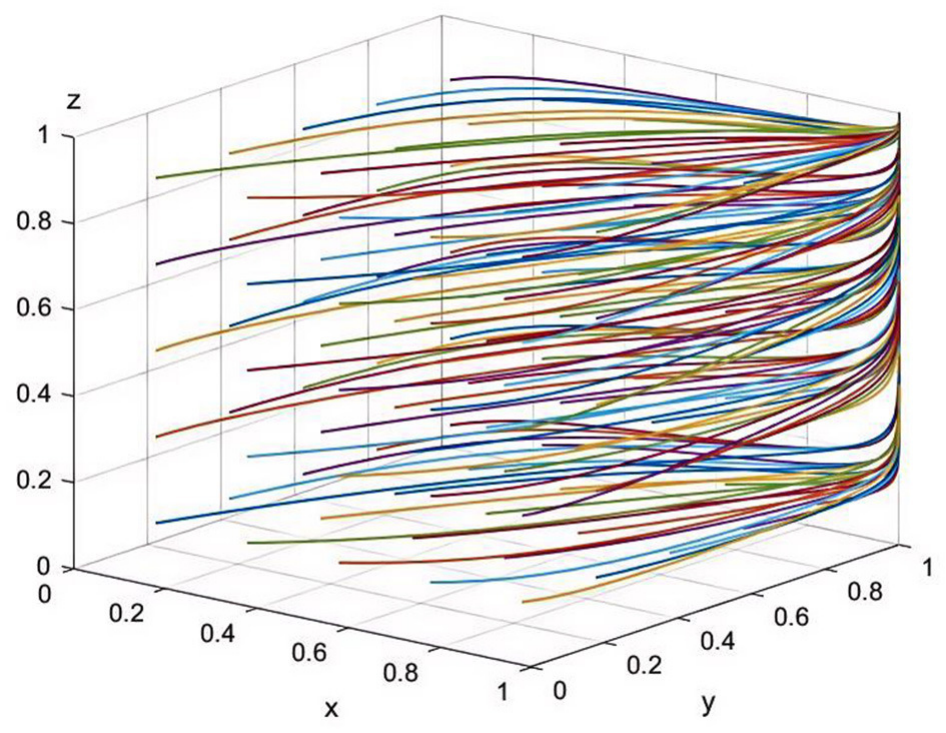
Simulation of parameter sensitivity at equilibrium point E8(1,1,1) (50 iterative simulations).

In the game model of this study, the system's stable equilibrium point, *E*_8_(1, 1, 1), represents an ideal state where pharmaceutical companies choose to produce and sell drugs at reasonable prices, healthcare institutions provide high-quality services, and the health insurance regulator enforces strict oversight. This equilibrium point is currently the optimal strategic choice and plays a crucial role in enhancing societal welfare (Figure 1). Initial strategy proportions were set as follows: *x* = 0.3, *y* = 0.4, *z* = 0.5, indicating that initially 30% of pharmaceutical companies adopt reasonable pricing strategies, 40% of healthcare institutions choose to provide high-quality services, and 50% of the health insurance regulators adopt strict oversight. These proportions reflect the transitional and mixed nature of current behavior observed during China's ongoing healthcare reform.

Firstly, the rational pricing strategy of pharmaceutical companies maintains a fair market competition environment and ensures the quality and safety of drugs, which positively impacts public health and the long-term development of the companies. Secondly, the high-quality services offered by healthcare institutions improve patient treatment outcomes and satisfaction, enhance their social influence and competitive market position, and provide a strong guarantee for public health. Lastly, the stringent regulation by the health insurance body effectively prevents irregular medical and pharmaceutical sales practices, driving continual improvements in the quality of services and products provided by healthcare institutions and pharmaceutical companies, ensuring that the public has access to fairly priced drugs and high-quality medical services.

Therefore, the equilibrium point *E*_8_(1, 1, 1) maximizes the interests of pharmaceutical companies, healthcare institutions, and the health insurance regulator, while also safeguarding public interests and societal welfare to the greatest extent. It reflects the balanced relationship among the three parties in medical reform and points toward the ideal goals of China's healthcare reform.

In the simulation process, the government fine for pharmaceutical companies *F*_*m*_ is set in the range of 500,000–3,000,000 RMB, aligning with the “Regulations on the Implementation of the Drug Administration Law of the People's Republic of China (amended in December 2024)” which stipulates a penalty of 2–5 times the illegal income for price violations. The initial value of *F*_*m*_ is set at the median of 1,500,000 RMB to reflect a typical enforcement level observed in practice.

### 4.2 Parameter analysis

The parameter sensitivity analysis aims to explore how changes in key variables affect the strategic choices and stability of the system. In MATLAB, we modify one parameter at a time—such as penalties, costs, or revenue—to observe how the strategic behavior of the three players responds. This one-variable-at-a-time technique helps isolate the influence of each factor while holding others constant. The results are visualized through line charts, allowing readers to intuitively grasp how system equilibrium shifts under different policy or economic scenarios.

#### 4.2.1 Sensitivity analysis of variables related to pharmaceutical companies

**Figure 2:**
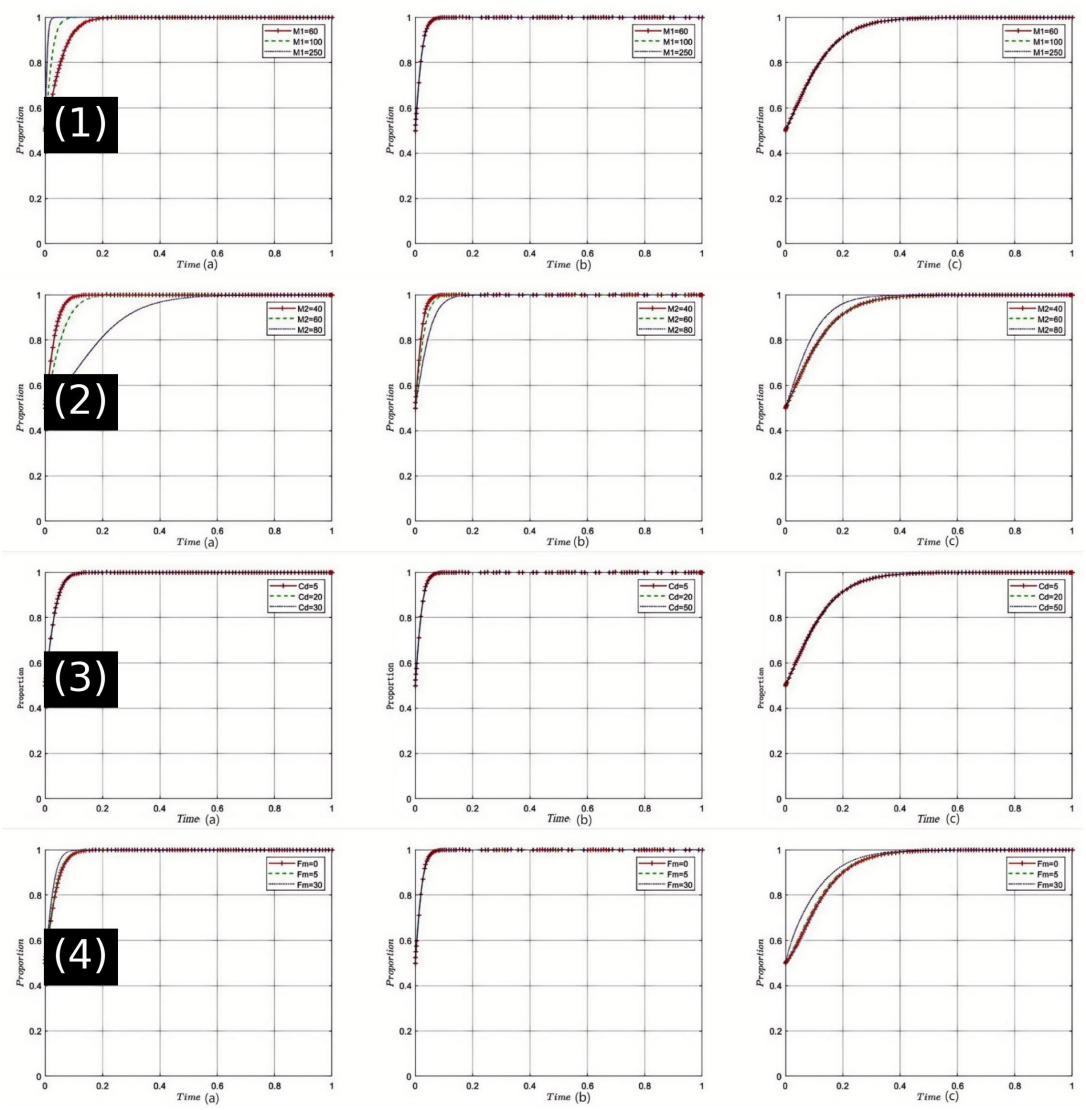
Comprehensive sensitivity analysis of key variables for pharmaceutical companies. (1) Sensitivity analysis of legitimate drug sales revenue (*M*_1_); (2) Sensitivity analysis of illegitimate drug sales revenue (*M*_2_); (3) Sensitivity analysis of research and development costs for pharmaceutical companies (*C*_*d*_); (4) Sensitivity analysis of government fines on pharmaceutical companies (*F*_*m*_).

Sensitivity Analysis of Rational Drug Sales Revenue (*M*_1_): The graphical results from the sensitivity analysis [Figure 2(1)] indicate that as *M*_1_ increases, pharmaceutical companies are more inclined to produce and sell drugs at reasonable prices. Additionally, as the rational sales revenue increases, healthcare institutions perceive an increase in the supply of reasonably priced drugs, thereby facilitating the provision of high-quality medical services. Concurrently, the health insurance regulator perceives the reasonableness of drug costs, bolstering its confidence in drug price regulation, thus making the enforcement of stringent regulatory strategies more feasible. In China's current healthcare system, drug pricing remains a core concern for both the public and government. High drug prices often lead to increased economic burdens for patients. Therefore, increasing *M*_1_ holds significant strategic importance for enhancing fairness and efficiency within the healthcare system.

Sensitivity Analysis of Unreasonable Drug Sales Revenue (*M*_2_): Numerical simulations [Figure 2(2)] show that an initial increase in *M*_2_ may encourage pharmaceutical companies to engage in unreasonable drug sales. However, as *M*_2_ continues to increase, this tendency gradually diminishes. Under these circumstances, healthcare institutions may face higher drug procurement costs, which could impact the quality of their services. The health insurance regulator might feel the pressure of increased payments, leading to stricter regulation of drug prices. In China's healthcare system, the phenomenon of exorbitant drug prices not only affects public access to healthcare but also leads to significant financial pressures on the health insurance fund. Thus, regulating *M*_2_ can help reduce such unreasonable sales practices and promote the healthy operation of the overall healthcare system.

Sensitivity Analysis of Research and Development Costs for Pharmaceutical Companies (*C*_*d*_): The simulation results [Figure 2(3)] illustrate that an increase in *C*_*d*_significantly impacts the production and sales strategies of pharmaceutical companies. Higher R&D costs may lead pharmaceutical companies to opt for less rational sales strategies in the short term. healthcare institutions might face drug supply shortages under these conditions, which could affect the quality of their services. Health insurance departments may also face increased pressures on drug payments. However, within the context of China's healthcare reforms, sustained investment in R&D is crucial for ensuring drug innovation, which is fundamental to enhancing the quality of healthcare services. Therefore, maintaining a reasonable level of *C*_*d*_ is vital for balancing short-term and long-term interests.

Sensitivity Analysis of Government Fines on Pharmaceutical Companies (*F*_*m*_): The analysis clearly shows [Figure 2(4)] that as *F*_*m*_ increases, pharmaceutical companies significantly reduce their engagement in irrational drug sales practices. Consequently, healthcare institutions benefit from more reasonable drug prices, which in turn allows them to offer higher quality services. The payment pressure on health insurance departments is accordingly reduced, enhancing their regulatory capabilities. In the current Chinese healthcare context, an effective fine mechanism is key to ensuring that pharmaceutical companies comply with regulations, and strict enforcement is a crucial component of ensuring fairness and sustainability in the healthcare system. Therefore, appropriately set *F*_*m*_ provides a robust guarantee for the stability of the healthcare system.

#### 4.2.2 Sensitivity analysis of variables related to healthcare institutions

**Figure 3:**
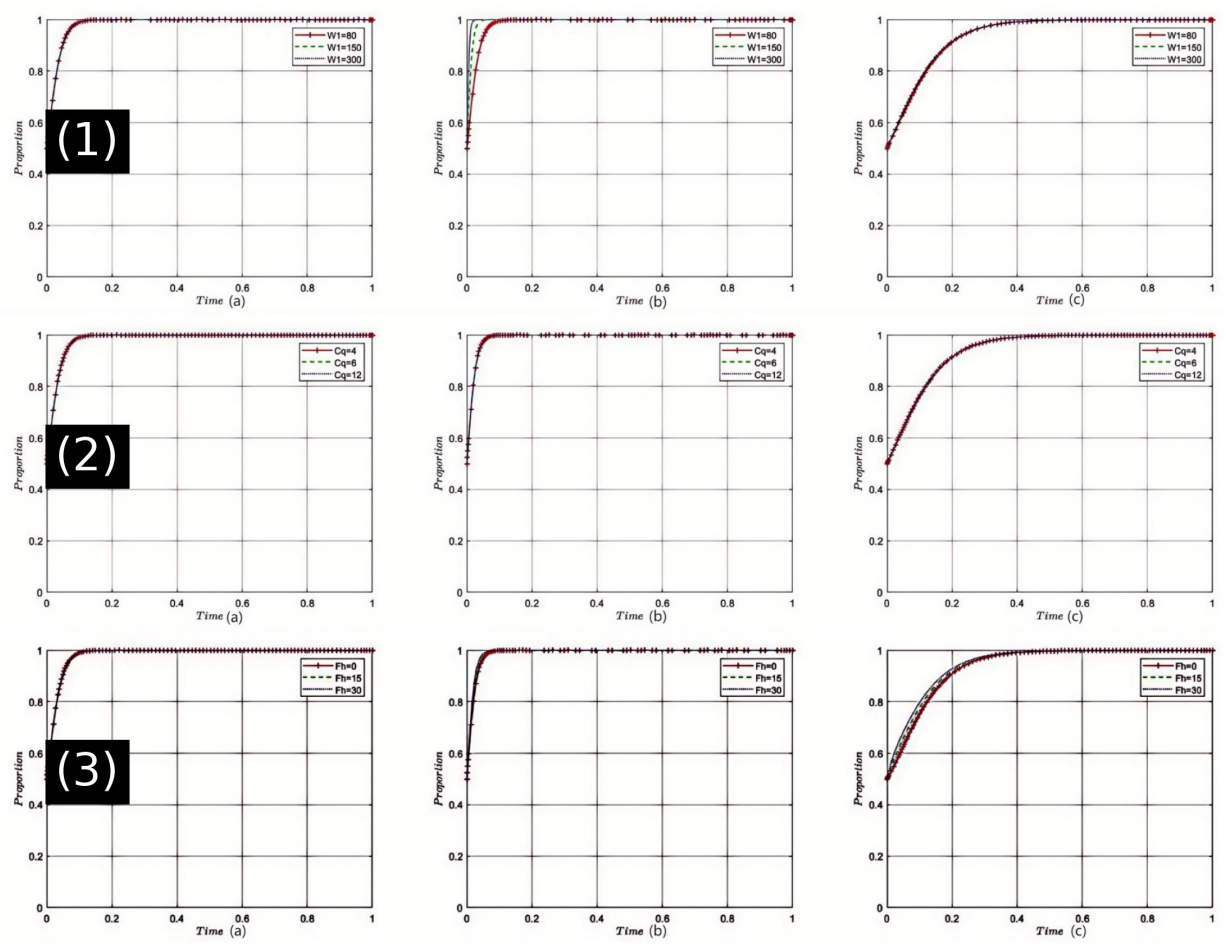
Comprehensive sensitivity analysis of key variables for Healthcare Institutions. (1) Sensitivity analysis of high-quality medical service revenue (*W*_1_); (2) Sensitivity analysis of service quality costs (*C*_*q*_); (3) Sensitivity analysis of fines imposed by health insurance departments on Healthcare Institutions (*F*_*h*_).

Sensitivity Analysis of High-Quality Medical Service Revenue (*W*_1_): The sensitivity analysis depicted in Figure 3(1), shows that as *W*_1_ increases, healthcare institutions are more inclined to provide high-quality medical services. Under this dynamic, pharmaceutical companies adapt to market changes by tending toward the production and sale of reasonably priced drugs. Health insurance departments, benefiting from an increase in high-quality medical services, reduce unnecessary expenditures, thereby strengthening the regulation of both drug and service quality. All parties are thus nudged closer to the optimal strategy choice at equilibrium point *E*_8_(1, 1, 1). Given the current state of China's healthcare system and the public's growing expectations for medical quality, enhancing *W*_1_ can stimulate healthcare institutions to improve service quality, thereby advancing the overall healthcare industry toward betterment.

Sensitivity Analysis of Service Quality Costs (*C*_*q*_): As shown in Figure 3(2), when *C*_*q*_ increases, healthcare institutions face heightened cost pressures, which may hinder their willingness to provide high-quality services. However, in the strategic game, once a certain threshold is exceeded, healthcare institutions might shift back toward offering high-quality services to mitigate risks, prompting pharmaceutical companies to adjust their strategies. Health insurance departments might intensify quality oversight to alleviate payment pressures, moving the tripartite game closer to equilibrium *E*_8_(1, 1, 1). Considering the backdrop of healthcare reform in China, controlling the cost of service quality is crucial as it impacts the operational efficiency and service level of healthcare institutions.

Sensitivity Analysis of Fines Imposed by Health Insurance Departments on healthcare institutions (*F*_*h*_): Simulation results [Figure 3(3)] indicate that with an increase in *F*_*h*_, healthcare institutions increasingly avoid non-compliance, returning to high-quality service provision. This shift influences pharmaceutical companies to more frequently opt for reasonably priced drugs, aligning with the changing demands of healthcare institutions. Health insurance departments may shift toward reward strategies, reducing penalties, and fostering a virtuous cycle. Under these conditions, the tripartite game gravitates more toward the choice of *E*_8_(1, 1, 1). In China's healthcare sector, a strict penalty mechanism can ensure compliance by healthcare institutions, but care must be taken not to overly suppress them to prevent other negative effects.

#### 4.2.3 Sensitivity analysis of variables related to health insurance regulator

**Figure 4:**
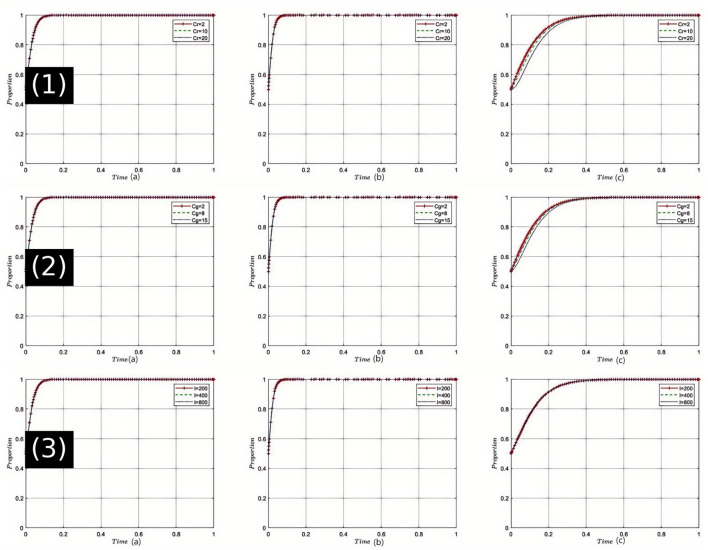
Comprehensive sensitivity analysis of key variables for health insurance regulator. (1) Sensitivity analysis of health insurance department's rectification costs (*C*_*r*_); (2) Sensitivity analysis of health insurance department's regulatory costs (*C*_*g*_); (3) Sensitivity analysis of health insurance fund revenue (*I*).

Sensitivity Analysis of Health Insurance Department's Rectification Costs (*C*_*r*_): The sensitivity analysis, as shown in Figure 4(1), indicates that with an incremental increase in *C*_*r*_, the health insurance department faces greater pressure during medical non-compliance rectification efforts. Elevated rectification costs may initially make the department more cautious, but once these costs exceed a certain threshold, the drive to rectify may be compromised. In this dynamic, pharmaceutical companies and healthcare institutions might anticipate a reduction in regulatory intensity, thereby seeking ways to circumvent oversight. However, if all parties can effectively cooperate and communicate to reduce rectification costs, achieving the equilibrium state *E*_8_(1, 1, 1) becomes more feasible. Considering the current state of healthcare in China, the rise in rectification costs could threaten the deepening of healthcare reform, making it urgent to find ways to reduce these costs.

Sensitivity Analysis of Health Insurance Department's Regulatory Costs (*C*_*g*_): As depicted in Figure 4(2), an increase in *C*_*g*_ necessitates more resources for health insurance regulation, which may impact the efficiency of oversight. During the evolution of the game, healthcare institutions and pharmaceutical companies might perceive a weakening in regulatory intensity and adjust their strategies to pursue higher profits. However, once all stakeholders recognize that excessive regulatory costs can deteriorate the medical environment, efforts to find a balance that leads to game outcomes approaching *E*_8_(1, 1, 1) will intensify. In the context of China's healthcare reforms, effectively controlling regulatory costs while ensuring effective oversight remains a critical challenge.

Sensitivity Analysis of Health Insurance Fund Revenue (I): Simulation results illustrated in Figure 4(3), show that as the health insurance fund revenue I increases, the health insurance department gains greater leeway in fund operations and protection. This growth strengthens its position in the game, making healthcare institutions and pharmaceutical companies more reliant on the support of the health insurance department. Consequently, both are more likely to collaborate with the department to facilitate the realization of *E*_8_(1, 1, 1). Given the realities of healthcare in China, including societal development and an aging population, the robust management of the health insurance fund is crucial, impacting not only individual health but also national stability.

### 4.3 Reflection on agent heterogeneity

While this study adopts a tripartite evolutionary game model assuming homogeneous behavior among pharmaceutical companies, healthcare institutions, and health insurance regulators, we recognize that real-world healthcare systems—especially in China—are far more heterogeneous. Institutions vary widely not only by geographic region but also by administrative tier. For example, a well-resourced tertiary hospital in a metropolitan area may respond quite differently to policy incentives or penalties compared to a rural township clinic with limited capacity. Similarly, pharmaceutical companies differ in compliance capabilities and strategic flexibility, while local health insurance regulators face varying fiscal and regulatory pressures. These disparities inevitably influence how stakeholders respond to policy reforms such as volume-based procurement or DRG-based payments. Although the current model abstracts these differences for analytical tractability, future extensions could reflect such heterogeneity by tuning key parameters—for instance, differentiating cost structures, penalty intensities, or subsidy levels across agent types. This would allow for a more nuanced understanding of reform outcomes under diverse institutional contexts and strengthen the model's applicability for region-specific policy design.

## 5 Results

Since the initiation of the “Triple Medical Reform” in China, the strategic interactions among health insurance departments, healthcare institutions, and pharmaceutical companies have increasingly become central to driving the efficiency of the healthcare system. The study results reveal complex dynamic interactions among the strategies of stakeholders in China's healthcare reform, with optimal strategies converging at equilibrium points. Specifically, pharmaceutical companies aim to maximize economic gains through drug pricing and quality control. Simulations show that high penalties (*F*_*m*_) for non-compliance significantly reduce unreasonable drug sales, and as reasonable drug sales revenue (*M*_1_) increases, companies are more inclined to adopt compliant strategies, thereby enhancing the overall stability of the healthcare system. Simultaneously, healthcare institutions strive to improve service efficiency and quality to meet patient needs. The study finds that increased subsidies (*S*) and high-quality service revenue (*W*_1_) significantly motivate institutions to improve service quality, while the costs of maintaining high-quality services (*C*_*q*_) and penalties from health insurance regulator (*F*_*h*_) influence the institutions' strategic decisions. Numerical simulations demonstrate that the system's sensitivity to various parameters, such as increasing reasonable drug sales revenue (*M*_1_) and high-quality service revenue (*W*_1_), contributes to system stability and compliance.

In the context of healthcare reform, health insurance regulatory agencies play a crucial role in ensuring the efficiency and fairness of fund utilization. The results indicate that higher income (*I*) and increased regulatory costs (*C*_*g*_) necessitate stricter oversight and management. Effective reward (*Y*_*b*_) and penalty mechanisms (*F*_*h*_) are vital for maintaining compliance among healthcare institutions and pharmaceutical companies. The system's stable equilibrium point (*E*_8_(1, 1, 1) represents an ideal state where pharmaceutical companies choose reasonable pricing, healthcare institutions provide high-quality services, and health insurance regulatory agencies enforce strict oversight. This equilibrium point maximizes the interests of all stakeholders and promotes social welfare. Initially, higher regulatory costs (*C*_*g*_) reduce oversight efficiency but eventually encourage stakeholders to cooperate more effectively, achieving the desired balance.

Additionally, the study incorporates real-world cases and data, such as the impact of centralized drug procurement and zero-markup policies, to validate the model's predictions. The results show significant improvements in drug affordability and service quality post-reform. Specifically, pharmaceutical companies should adopt reasonable pricing and quality control strategies to ensure long-term industry stability and societal trust while pursuing economic benefits. Healthcare institutions should enhance service efficiency and quality through efficient operational management to meet patient demands and increase competitiveness and market share. Health insurance regulatory agencies should maintain fund utilization efficiency and fairness through meticulous policy formulation and implementation, ensuring the sustainability of the health insurance system. These coordinated strategies are critical for driving China's healthcare system toward greater efficiency and equity. Based on the current research findings, pharmaceutical companies should continually optimize drug research and development and market strategies. healthcare institutions need to further enhance service efficiency and quality, while health insurance regulatory agencies should intensify regulatory efforts, promote policy transparency and fairness, and collectively build a more stable and efficient healthcare environment.

## 6 Discussion

The strategic interplay among pharmaceutical companies, healthcare institutions, and health insurance regulator is crucial for the success of healthcare reform. Only through mutual strategic collaboration and joint efforts can these stakeholders drive the Chinese healthcare system toward more efficient and equitable medical services.

### 6.1 Strategic interplay among stakeholders

Pharmaceutical companies need to strengthen their collaboration with healthcare institutions and health insurance departments to ensure drug quality and stable supply. They must engage in effective negotiations to maintain and enhance their position in healthcare reform. As the primary providers of pharmaceuticals, their strategic choices significantly impact the stability and efficiency of the entire healthcare system. Continuous investment in research and development, enhancement of drug innovation, and exploration of reasonable pricing mechanisms are essential for ensuring drug quality and market competitiveness.

### 6.2 Policy implications and enhanced collaboration

Healthcare institutions play a pivotal role as frontline service providers. They must actively participate in negotiations with health insurance departments and form collaborations with pharmaceutical companies to jointly advance healthcare reform. Enhancing service levels is crucial for improving patient experience and treatment outcomes. Establishing stable collaborations with pharmaceutical companies and engaging in in-depth negotiations with health insurance departments promote the integration of drugs and medical services. Policy-makers should actively encourage enhanced collaboration among pharmaceutical companies, healthcare institutions, and health insurance regulatory agencies by promoting joint initiatives such as research collaborations and quality improvement projects.

### 6.3 Transparency, information sharing, and capacity building

Transparency and information sharing among stakeholders are essential for aligning strategies and improving overall efficiency. Policy-makers should ensure that all stakeholders have access to relevant information, leading to more informed decision-making and improved coordination. Investing in capacity building for all stakeholders involved in the healthcare system is crucial. This includes providing training and resources to pharmaceutical companies, healthcare institutions, and health insurance regulatory agencies to improve their capabilities in adapting to and implementing reforms. Enhanced transparency and capacity building are vital for driving the Chinese healthcare system toward greater efficiency and equity.

### 6.4 Real-world implications and future research directions

Our study suggests that coordinated efforts among pharmaceutical companies, healthcare institutions, and health insurance regulator can lead to more affordable and accessible healthcare services. Reasonable drug pricing and quality control can make medications more affordable, and improved service quality can enhance patient outcomes. Strict oversight can ensure efficient health insurance fund utilization, improving coverage and reimbursement rates. However, this study has certain limitations, including potential oversimplification of the complex dynamics among stakeholders and the exclusion of other potential stakeholders such as patients and medical staff. Future research should aim to refine the model to capture these complexities more comprehensively and consider the perspectives of a broader range of stakeholders. This will provide a more robust theoretical and practical foundation for advancing healthcare reform and enhancing public health welfare.

Compared to existing studies that focus on single-sector or bilateral regulatory models, such as insurer–hospital or pharma–provider relationships, our findings demonstrate that a tripartite coordination mechanism significantly reduces systemic instability and enhances the alignment of stakeholder incentives. For instance, while bilateral models often face coordination failures due to asymmetric information or conflicting objectives, the inclusion of a third-party regulator in our model introduces dynamic checks and balances, enabling more stable evolutionary paths and convergence to socially optimal strategies. This highlights the comparative advantage of multi-agent strategic coordination in complex healthcare systems, particularly in the context of China's comprehensive reforms. Recent regional reforms in China demonstrate that a coordinated tripartite approach can significantly reduce system instability and improve healthcare efficiency. The centralized drug procurement policy in Shanghai has led to substantial reductions in drug prices and enhanced medication accessibility, while DRG payment pilots in Beijing have improved cost control and streamlined service delivery. These outcomes provide concrete evidence that the multi-agent coordination model developed in this study can effectively inform policy decisions and enhance public health outcomes.

## 7 Conclusion

In this study, the strategic interactions among pharmaceutical companies, healthcare institutions, and health insurance regulator under the context of healthcare reform in China were extensively explored using a game theory model. This revealed their pivotal roles and strategic choices in promoting the efficiency and equity of the healthcare system. The research indicates that when these three key stakeholders adopt coordinated strategies—pharmaceutical companies committing to reasonable pricing and quality control, healthcare institutions striving to improve service quality and efficiency, and health insurance regulator enforcing strict oversight—an equilibrium at point *E*_8_(1, 1, 1) can be achieved. At this state, not only are the interests of all parties maximally protected, but the entire healthcare system also becomes more equitable and efficient. Therefore, based on the actual needs of healthcare reform and its future direction, it is recommended to enhance communication and cooperation among these parties and optimize the formulation and implementation of related policies, thus effectively deepening healthcare reform and enhancing public health welfare.

However, this study also has the following limitations: Firstly, although the game theory model used can reflect the basic strategic interactions among the three parties, it may not capture the full complexity and nuances of actual operations. Secondly, given the limitations of research resources and data, other potential stakeholders affecting healthcare reform, such as patients and medical staff, have not yet been included. The current model still needs further refinement and enhancement to more comprehensively capture the complex dynamics and diverse interests in healthcare reform, providing a more robust theoretical and practical foundation for future research.

## Data Availability

The original contributions presented in the study are included in the article/supplementary material, further inquiries can be directed to the corresponding author.
